# Dr. Carpenter's Reply to Mr. Wharton Jones, on the Functions of the White Corpuscles

**Published:** 1844-10

**Authors:** W. B. Carpenter

**Affiliations:** Ripley


### ON THE FUNCTIONS OF THE WHITE CORPUSCLES. BY DR. CARPENTER.

*To the Editor of the British and Foreign Medical Review.*

MY DEAR SIR,—It is not my intention to request a place in your Review for any lengthened reply to the arguments by which Mr. Wharton Jones has attempted to subvert my views on the functions of the White Corpuscles, and has defended his own previously expressed doctrines; for I think that its pages may be much better occupied than with a voluminous discussion of this kind, from which each party will be pretty sure to retire, with an increased conviction of the truth of his own views, and of the weakness of his adversary's position. But I must request the attention of your readers to the manner in which Mr. Wharton Jones disposes of one of my chief arguments,—the *absence* of anything resembling the *red* corpuscles, in the blood of invertebrated animals; and the universal *presence* of floating cells, exactly resembling the *colourless* corpuscles of the blood of vertebrata, in greater or less abundance. On this Mr. W. J. contents himself with remarking (§ 62) that, "considering how very imperfect the knowledge of the blood of the invertebrata is as yet, any analogical argument drawn from it in the present case is of too loose a nature to merit the slightest attention." This is certainly a very convenient manner of answering a troublesome argument; but I shall endeavour to show that it is quite inadmissible in the present instance.

I think that no unprejudiced person can refuse assent to the proposition, that the processes of nutrition must be essentially the same in the invertebrata as in vertebrated animals: their food is the same, and must yield albumen; their blood coagulates, and must therefore contain fibrin; and their tissues have the same microscopical and chemical characters, and may therefore be presumed to originate in the same manner. The chief difference in their nutritive apparatus consists in the absence of any *special* absorbing system; and in the low amount of heat generated by them, except in the class of insects under particular circumstances—for which the peculiar arrangement of their respiratory apparatus fully accounts, as I have shown on a former occasion.

That the blood or circulating fluid of the invertebrata does not contain red corpuscles, and that both in the character of its floating cells, and its imperfect coagulation, it rather resembles the chyle and lymph of vertebrata, was first, I believe, stated by Wagner; and it was on his authority that I introduced the fact into my Report. But during a stay of some weeks by the sea-side last autumn, I had the opportunity of verifying the statement upon a large number of minute annelida, crustacea, and mollusca, in which I could watch the circulation during life; and I have at other times made many similar observations upon the transparent aquatic larvæ of insects. The fact may, I think, be regarded, therefore, as tolerably well established.

Now if we apply the term blood to the nutritious fluid of animals in general, it is evident that the presence of *red* corpuscles, being *limited* to one only of the four sub-kingdoms, cannot be regarded as essential to the character of this fluid; and their function must be one *peculiar* to the group in which they appear. On the other hand, the presence of those floating cells, to which we give the name of *colourless* corpuscles, appears to be *universal;* and their function must be one of a *general* kind. That their use is not to generate red corpuscles is evident from the total absence of the latter. That if either kind of corpuscles be the agents in elaborating the plastic element of the blood, it must be the *white,* appears evident from the same consideration,—this being the most universal of all functions, without which no tissue can be generated. On the other hand, that the *red* corpuscles are subservient to the generation of *heat,*—a function limited to vertebrata, with the exception already noticed,—appears from the constant proportion which exists under ordinary circumstances between the number of red corpuscles in the blood, and the heat developed in each tribe of animals,—a general doctrine which receives important confirmation from recent experiments of Dr. John Davy (Philosophical Transactions, 1844.)

My argument, therefore, is not an *analogical* but a *direct* one; and seems to me to bear more closely on the question at issue than any which has been urged. If fibrin can be elaborated without *red* corpuscles, they cannot be the agents of its production. If, on the other hand, *white* corpuscles present themselves wherever fibrin is in process of elaboration (and I have yet to learn that there is any exception to this generalization), there seems a strong probability in favour of their connexion with the process.

Mr. Wharton Jones seems, by his note to § 45 of the first part of his Report, to think that I have deprived him of his due in placing Wagner and Henle on a level with himself as the enunciators of the doctrine, that the fibrin of the blood is elaborated from the albumen by the agency of the red corpuscles. I can only say that I had fully comprehended this to be Wagner's view, from the perusal of Dr. Willis's translation of his 'Physiology' (p. 448), before the publication of Mr. Wharton Jones's Report on the Blood; nor can I now see how the expression,—that the red corpuscles "bear the same relation to the plasma and its normal composition as the cellular parts of the secreting glands do to the secreted fluids,"—is to be understood in any other manner. Believe me to be, my dear Sir, yours most truly, W. B. Carpenter.

Ripley; *Sept.* 12*th,* 1844.

---

